# Facile and green reduction of covalently PEGylated nanographene oxide via a ‘water-only’ route for high-efficiency photothermal therapy

**DOI:** 10.1186/1556-276X-9-86

**Published:** 2014-02-18

**Authors:** Jingqin Chen, Xiaoping Wang, Tongsheng Chen

**Affiliations:** 1 MOE Key Laboratory of Laser Life Science and Institute of Laser Life Science, College of Biophotonics, South China Normal University, Guangzhou 510631, People’s Republic of China; 2 Department of Pain Management, the First Affiliated Hospital of Jinan University, Guangzhou 510632, People’s Republic of China

**Keywords:** Graphene oxide, Covalent functionalization, Green reduction, Near-infrared absorbance, Bioapplication, Photothermal therapy

## Abstract

A facile and green strategy is reported for the fabrication of nanosized and reduced covalently PEGylated graphene oxide (nrGO-PEG) with great biocompatibility and high near-infrared (NIR) absorbance. Covalently PEGylated nGO (nGO-PEG) was synthesized by the reaction of nGO-COOH and methoxypolyethylene glycol amine (mPEG-NH_2_). The neutral and purified nGO-PEG solution was then directly bathed in water at 90°C for 24 h without any additive to obtain nrGO-PEG. Covalent PEGylation not only prevented the aggregation of nGO but also dramatically promoted the reduction extent of nGO during this reduction process. The resulting single-layered nrGO-PEG sheets were approximately 50 nm in average lateral dimension and exhibited great biocompatibility and approximately 7.6-fold increment in NIR absorption. Moreover, this facile reduction process repaired the aromatic structure of GO. CCK-8 and flow cytometry (FCM) assays showed that exposure of A549 cells to 100 μg/mL of nrGO-PEG for 2 h, exhibiting 71.5% of uptake ratio, did not induce significant cytotoxicity. However, after irradiation with 808 nm laser (0.6 W/cm^2^) for 5 min, the cells incubated with 6 μg/mL of nrGO-PEG solution showed approximately 90% decrease of cell viability, demonstrating the high-efficiency photothermal therapy of nrGO-PEG to tumor cells *in vitro*. This work established nrGO-PEG as a promising photothermal agent due to its small size, great biocompatibility, high photothermal efficiency, and low cost.

## Background

Graphene oxide (GO), a low-cost carbon nanomaterial synthesized from graphite with fascinating physical and chemical properties, has been widely developed in the past several decades [1-4]. Its unique nanostructure holds great promise for potential applications in biomedicine such as microbial detection, nanocarrier systems, and photosensitizer [5-9]. However, GO is unstable and tends to aggregate in water due to the Van der Waals interaction and strong *π*-*π* stacking between GO nanosheets. To render superior stability in aqueous solution, GO is generally ultrasonicated to nanosized GO (nGO) and then functionalized covalently by biocompatible and nontoxic surfactant such as polyethylene glycol (PEG) and p-aminobenzenesulfonic acid via amido bond [10-12]. Covalently PEGylated nGO (nGO-PEG) can be used as nanocarriers for aromatic antitumor drugs such as doxorubicin (DOX), SN38, and camptothecin (CPT) through *π*-*π* stacking between drugs and nGO surface [8,13,14]. Recently, nGO-PEG has also been used as photoabsorbing agent for photothermal therapy (PTT) [15-17]. However, the very low near-infrared (NIR) absorption of nGO-PEG leads to the inefficient ablation of large tumors or tumors deeply located inside the body and the usage of relatively high NIR laser power for the PTT of nGO-PEG [2,15-19].

The reduced nGO (nrGO) has attracted great interest especially in PTT [20-24] since the reduction of nGO by removing oxygen-containing groups can result in a dramatic increment in NIR absorption. Toxic chemical reducing reagents, such as sodium borohydride, hydrazine, and its derivatives, are usually used to reduce nGO. Although the nrGO produced by chemical reduction methods have high conductivity and C/O ratio, the intense agglomeration and the residual toxic reduction reagent limit its bioapplication [20,21,25,26]. Recently, some green strategies have been developed to produce soluble nrGO. Natural extracts like green tea, gelatin, and spinach leaf can act as both the green reduction reagents and functionalization reagents [23,27-29]. Strong alkaline and alcohols were also utilized to reduce nGO [30,31]. In addition, a ‘water-only’ green reduction route to produce graphene by hydrothermal dehydration under high temperature of 180°C was reported, in which the overheated supercritical (SC) water in sealed container acted as reducing agent [32].

Herein, we developed a more facile and green strategy to obtain stable nrGO-PEG by reducing nGO-PEG in water. In addition, to the ability to recover *π*-conjugation via repairing the postreduction defects, our approach has three marked advantages over the previously reported green reduction processes: (1) the reduction process requires very simple setup and low temperature, that is, basically a water bath kettle, (2) the resulting nrGO-PEG exhibits great biocompatibility and noncytotoxicity, making it a promising candidate for biorelated application, (3) the resulting nrGO-PEG has an approximately 7.6-fold increment in NIR absorption, making nrGO-PEG a potential photosensitizer for PTT.

## Methods

### Synthesis of nGO-PEG

The nGO-PEG was synthesized following previous studies in our laboratory [13,16]. In brief, GO was made by a modified Hummer's method utilizing expandable graphite flake (XF NANO Co., Ltd., Nanjing, China) [33,34]. nGO solution was prepared by sonication of GO flake. NaOH (1.2 g) and Cl-CH_2_-COOH (1.0 g) were added to nGO suspension (approximately 2 mg/mL) and sonicated for 30 min to obtain carboxylation nGO (nGO-COOH). The resulting nGO-COOH suspension was neutralized and purified by repeatedly rinsing and filtration. Methoxypolyethylene glycol amine (5 kDa MW, mPEG-NH_2_, PEG BIO Co., Ltd., Suzhou, Jiangsu, China) and nGO-COOH suspension reacted at pH of 6 and then, 1-ethyl-3-(3-dimethylaminopropyl)carbodiimide (4 mM) and N-hydroxysuccinimide (10 mM) (EDC and NHS, Sigma, St. Louis, MO, USA) were added to the above suspension. The nGO-PEG was purified by centrifuging with 30 kDa ultracentrifuge tube (Millipore, Billerica, MA, USA) and dialyzed against distilled water for overnight to remove any ions, and then, by centrifuging the solution of the mixture at 12,000× *g* for 30 min to remove any unstable aggregates.

### Synthesis of nrGO-PEG

Twenty milliliters of nGO-PEG solution (approximately 0.5 mg/mL) was transferred to a sealed glass bottle and then bathed at different temperature for 24 h or bathed at 90°C for different times. The resulting nrGO-PEG solution was centrifuged at 6,000× *g* for 30 min to remove any unstable aggregates and stored at 4°C for further use.

### Spectroscopic characterization

The GO, nGO, nGO-PEG, and nrGO-PEG sheets were imaged with atomic force microscopy (AFM, Agilent Technologies 5500, Santa Clara, CA, USA) on a mica substrate. UV-vis spectra were performed by a UV-vis spectrometer (Lambda 35, Perkin-Elmer, Waltham, MA, USA) with a 1-cm quartz cuvette. Fourier transform infrared (FTIR) spectra were recorded on a FTIR spectrometer (Bruker Tensor 27, Karlsruhe, Germany). Raman spectra were taken with a Renishaw (New Mills, UK) inVia micro-Raman spectroscopy system equipped with a 514.5-nm Ar^+^ laser. The images of all samples were recorded using a digital camera (Nikon, Tokyo, Japan) with 1,280 × 1,280 pixels resolution.

### Fluorescence labeling of nrGO-PEG and cell uptake assay

The nrGO-PEG was labeled by fluorescein isothiocyanate (FITC, Sigma). In brief, the solution of nrGO-PEG (approximately 0.5 mg/mL) was mixed with 0.1 mL FITC (13 mM) dissolved in DMSO and then stirred overnight at room temperature. The resulting mixtures labeled with FITC were filtrated through 30 kDa filters to remove excess unbound FITC and centrifuged at 12,000× *g* for 30 min to eliminate solid aggregated FITC. The obtained nrGO-PEG/FITC was re-dispersed in distilled water. The whole procedures were operated in the dark place.

A549 cells (1 × 10^5^ cells) were incubated with 100 μg/mL of nrGO-PEG/FITC and free FITC for 2 h, respectively, in the dark. After that, the cells were rinsed by phosphate buffered saline (PBS) five times. Fluorescence emission from FITC was observed using a confocal microscope (LSM 510/ConfoCor 2, Zeiss, Jena, Germany). FITC was excited at 488 nm laser with an Ar-Ion laser (reflected by a beam splitter HFT 488 nm), and fluorescence emission was recorded by a 505 to 550-nm IR band-pass filter. The uptake ratio of nrGO-PEG by A549 cells were measured by flow cytometry (FCM, FACSCantoII, Becton Drive, New Jersey, USA) using FITC labeled on nrGO-PEG, and for each FCM analysis, 10,000 events were recorded.

### Photothermal irradiation

The nrGO-PEG solution was diluted to a desired concentration of 3, 6, 10, 20, and 30 μg/mL with distilled water. The above samples, 3 μg/mL nGO-PEG, and distilled water were continuously irradiated by 808 nm NIR laser with the power density of 0.6 W/cm^2^ for 8 min. Temperature was measured by a thermocouple thermometer (Fluke 51II, Lake Mary, FL, USA) every other 30 s. In addition, the temperature of water, 3 μg/mL nGO-PEG, and nrGO-PEG solution after the irradiation were also measured by infrared thermal camera (TVS200EX, NEC, Minato, Tokyo, Japan). All the experiments were conducted at room temperature.

### Cell culture and cytotoxicity assay

A549 cell line obtained from the Department of Medicine, Jinan University (Guangzhou, China) was cultured in Dulbecco's modified Eagle's medium (DMEM, Gibco, Grand Island, NY, USA) supplemented with 10% fetal calf serum (FCS) in 5% CO_2_, 95% air at 37°C in a humidified incubator.

For cytotoxicity assay, approximately 5,000 A549 cells/well were plated in 96-well plate with 100 μL medium and cultured for 24 h, and then, various concentrations of nGO-PEG and nrGO-PEG were added to the wells. For photothermal therapy, adherent cells were incubated at various concentrations of nGO-PEG and nrGO-PEG for 2 h and then were irradiated by 808 nm semiconductor laser with the power density of 0.6 W/cm^2^ for 5 min. The relative cell viability was assessed by Cell Counting Kit-8 (CCK-8, Dojindo, Kamimashiki-gun, Kumamoto, Japan) assay as described previously [35] and determined by a 96-well plate reader (INFINITE M200, Tecan, Seestrasse, Männedorf, Switzerland) at an absorbance value of 450 nm. All experiments were performed in quadruple occasion.

### Cell death assay

A549 cells were cultured in 48-wells plate for 24 h and treated with different concentrations of nGO-PEG and nrGO-PEG for 2 h and then were irradiated by 808 nm semiconductor laser with the power density of 0.6 W/cm^2^ for 5 min. The living and dead cells in the medium were harvested and washed with PBS and then measured by FCM using AnnexinV-FITC/PI apoptosis detection kit (Bender Medsystems, Vienna, Austria) as previously described [35], and 10,000 events were recorded for each FCM analysis.

## Results and discussion

### Synthesis and characterization of nGO-PEG and nrGO-PEG

To make a well-dispersed and stable nGO solution with high NIR absorbance for bioapplication, we have developed a facile and green reduction method to prepare nrGO-PEG.

•Step 1: PEGylation of nGO. GO was obtained by oxidization of graphite according to the modified Hummers' method and sonication of GO, resulting in nGO [33]. PEGylation of nGO is usually performed to improve the stability of nGO under physiological conditions [8,36]. In the present strategy, mPEG-NH_2_ was introduced to covalently conjugate with nGO-COOH by chemical reaction between -NH_2_ groups and carboxyl groups under the participation of NHS and EDC, resulting in nGO-PEG [37].

•Step 2: green reduction of nGO-PEG. The stable neutral nGO-PEG solution was bathed in water at 90°C for 24 h.

**Figure 1:**
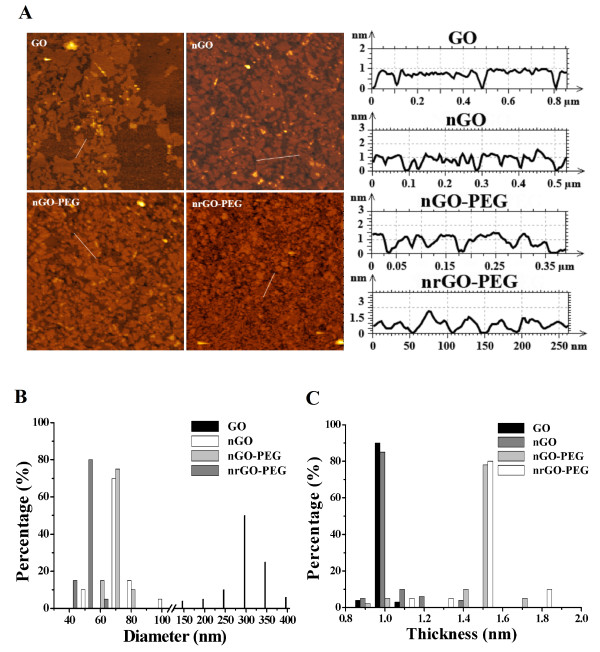
**AFM characterization. (A)** AFM images of GO, nGO, nGO-PEG, and nrGO-PEG. AFM histograms of **(B)** diameter and **(C)** thickness for GO, nGO, nGO-PEG, and nrGO-PEG. The diameter was assumed to be in round shape.

Figure 1A showed the AFM images of GO, nGO, nGO-PEG, and nrGO-PEG. GO, nGO, nGO-PEG, and nrGO-PEG presented 300, 68, 71, and 52 nm in sheet diameter (assuming that these arein round shape), respectively (Figure 1B). The sheet thickness of nrGO-PEG (approximately 1.5 nm) was similar to that of nGO-PEG but much higher than that of nGO and GO (approximately 1.0 nm) (Figure 1C), likely owing to the covalent PEGylation that offered more condensed surface polymer coating on nGO or to the existence of partial PEG on the reduced nGO surface [21,38,39]. Interestingly, nrGO-PEG was much smaller than nGO-PEG in lateral width (Figure 1B), likely due to the breakage of chemical bond between graphene sheets during reduction process.

**Figure 2:**
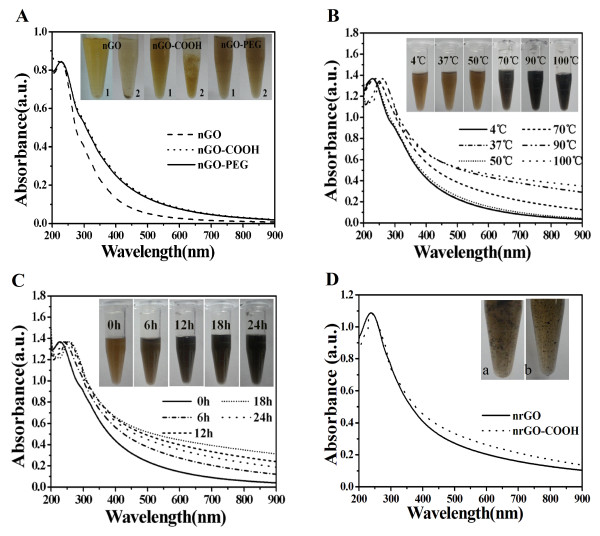
**Reduction of nGO-PEG. (A)** UV-vis spectra of nGO, nGO-COOH, and nGO-PEG suspension with approximately 15 μg/mL of GO (1 cm optical path). Inset: photographs of nGO, nGO-COOH, and nGO-PEG suspension at the same GO concentration (1) before and (2) after storage for a week at 4°C. **(B)** UV-vis absorption spectra of the nGO-PEG solution bathed at indicated temperature for 24 h. Inset: photographs of nGO-PEG at the indicated temperature for 24 h. **(C)** UV-vis absorption spectra of the nGO-PEG solution bathed at 90°C for various time. Inset: photographs of nGO-PEG bathed at 90°C for various time (0, 6, 12, 18, 24 h). **(D)** UV-vis spectra of (a) nrGO and (b) nrGO-COOH suspension bathed at 90°C for 24 h.

As seen in the UV-vis spectra of nGO, nGO-COOH, and nGO-PEG, the strong optical absorption peak at 230 nm and the weak shoulder at 300 nm, originating from the *π* → π* transitions of aromatic C = C bond and the *n* → π* transitions of C = O bond, remained essentially unchanged (Figure 2A). Compared with nGO, both nGO-COOH and nGO-PEG exhibited higher absorbance at long wavelength (more than 300 nm) (Figure 2A), which was further verified by the color change of nGO suspension from brown to black brown during carboxylation and PEGylation (Figure 2A inset 1), probably due to the partial and slight reduction of nGO under strong alkaline conditions [30,36]. The neutral nGO and nGO-COOH were dispersed at that time of preparation (Figure 2A inset 1) but aggregated and precipitated after storage for a week at 4°C (Figure 2A inset 2), most likely due to the screening of electrostatic charge on nGO and the *π*-*π* stacking between nGO sheets [8,40]. However, the nGO-PEG suspension remained stable for at least 3 months under the same conditions (Figure 2A inset 2), suggesting that covalent PEGylation enhanced the stability of nGO, which was benefited to its storage and bioapplication.

Next, we reduced nGO-PEG by transferring 20 mL nGO-PEG solution (approximately 0.5 mg/mL) to sealed glass bottles that were then bathed at 4°C, 37°C, 50°C, 70°C, 90°C, and 100°C respectively for 24 h. It was found that the color of nGO-PEG solution at 4°C, 37°C, and 50°C remained nearly unchanged but changed to black brown and dark black at 70°C, 90°C, and 100°C, respectively (Figure 2B inset), indicating the reduction reaction at the temperature range of 70°C to 100°C. The color change from brown to dark black was evident for the reduction of nGO [28,32]. Consistent to the color change, UV-vis spectra revealed that the nGO-PEG solution bathed at 70°C, 90°C, and 100°C, respectively, for 24 h showed approximately 3.1, approximately 7.6, and approximately 7.8-fold increment in NIR absorption at 808 nm (Figure 2B). Moreover, bathing at 70°C to 100°C for 24 h resulted in a red-shift of the peak at 230 to 261 nm (Figure 2B), indicating partial restoration of the *π*-conjugation network of carbon structure. In addition, disappearance of the weak shoulder peak at 300 nm (Figure 2B) reflected the effect of deoxygenation [28,32]. Although the nrGO-PEG suspension obtained at 100°C had an approximately 7.8-fold increment in NIR absorption, it showed some aggregations (Figure 2B inset), likely due to the complete removal of oxygen groups and amido bond of nGO-PEG [31,32]. Subsequently, we also evaluated the effect of reduction time at 90°C on the reduction extent and found that the color of nGO-PEG solution changed from black brown to dark black with increasing bathing time (Figure 2C inset), and UV-vis spectra showed that the nGO-PEG solution bathed for 24 h had higher NIR absorption than others (Figure 2C).

As control, we next assessed the effect of this reduction method on both nGO and nGO-COOH solutions. In brief, nGO and nGO-COOH solutions were bathed at 90°C for 24 h. UV-vis spectra showed that nGO and nGO-COOH had approximately 3.1 and approximately 3.4-fold increment in NIR absorbance (at 808 nm) (Figure 2D), which was further verified by the color change (darkening) of nGO and nGO-COOH solutions (Figure 2D inset). After reduction, the absorption peak (230 nm) of nGO and nGO-COOH red-shifted to approximately 238 nm and the shoulder peak (300 nm) nearly disappeared (Figure 2D). As shown in Figure 2A,D, carboxylation of nGO and carboxyl group did not affect the reduction extent of nGO. Although nGO and nGO-COOH could be reduced by this green reduction method (Figure 2D), the reduced nGO and nGO-COOH showed intense aggregations and relative low NIR absorbance (Figure 2B,C,D inset), indicating that the endogenous PEG chains of nGO-PEG remarkably strengthened the reduction extent of nGO during this facile and green reduction process.

**Figure 3:**
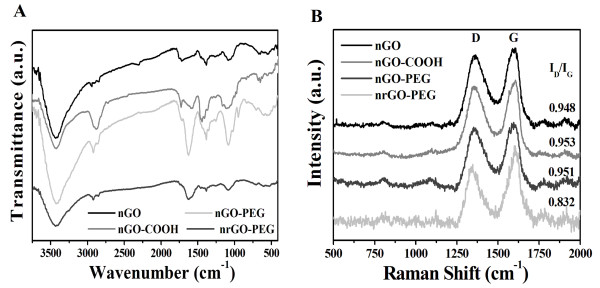
FTIR (A) and Raman spectra (B) of nGO, nGO-COOH, nGO-PEG, and nrGO-PEG.

FTIR spectrometer was used to further confirm the conjugation between PEG and nGO-COOH and the change of oxidation group of nrGO-PEG (Figure 3A). The presence of intense bands at around 3,400 cm^−1^, approximately 1,720 cm^−1^ (C = O), approximately 1,580 cm^−1^ (C = C), and approximately 1,204 cm^−1^ (C-O-C) indicated the existence of oxygen containing moieties such as carbonyl, carboxylic, epoxy, and hydroxyl in nGO. After carboxylation, nGO-COOH showed the presence of bands at approximately 2,880 cm^−1^ (−CH_2_−) and approximately 1,720 cm^−1^ (C = O). The existence of characteristic amide-carbonyl (−NH-CO–) stretching vibration (approximately 1,650 cm^−1^) in nGO-PEG indicated that nGO-COOH was covalently conjugated with mPEG-NH_2_ via amido bond successfully. Removal of oxygen-containing groups in all nrGO are clearly indicated by the disappearance of C = O stretching, C-O-C stretching, and C-O stretching bands and the relative decrease in the intensity of broad band at approximately 3,400 cm^−1^ for the hydroxyl group [28,29]. In addition, for nrGO-PEG, the C = O vibration band nearly disappeared and the remained broad –OH, –CH_2_–, −NH-CO–, and C-O-C stretching bands decreased considerably. It was clear that this water-only approach effectively reduced nGO-PEG to nrGO-PEG (Figure 2). Therefore, the existence of –OH, –CH_2_–, −NH-CO–, and C-O-C stretching bands may be contributed by the partial conjunct PEG on nrGO, which maybe the reason that nrGO-PEG was thicker than nrGO (Figure 1C).

Raman spectroscopy was used to characterize nGO, nGO-COOH, nGO-PEG, and nrGO-PEG (Figure 3B). The typical features in Raman spectra are the G band at 1,600 cm^−1^ and the D band at 1,350 cm^−1^. The G band is usually assigned to the E_2g_ phonon of C sp^2^ atoms [41], while the prominent D band is an indication of disorder corresponding to defects associated with vacancies, grain boundaries, and amorphous carbon species [42,43]. As shown in Figure 3B, the intensity ratio (*I*_D_/*I*_G_) of D band to G band of nGO, nGO-COOH, nGO-PEG, and nrGO-PEG were about 0.948, 0.953, 0.951, and 0.832, respectively. Obviously, nGO-COOH and nGO-PEG had similar *I*_D_/*I*_G_ value with that of nGO, indicating that both carboxylation and PEGylation did not destroy the aromatic structures of nGO. However, the *I*_D_/*I*_G_ value of nrGO-PEG was much lower than that of nGO, suggesting that the reduction reaction in this facile and green reduction strategy developed here was also able to recover the aromatic structures by repairing defects. It is reported that the *I*_D_/*I*_G_ ratio of chemically reduced nGO is higher than that of as-made GO due to the presence of unrepaired defects after the removal of oxygen moieties [25,31,44]. Therefore, the green reduction approach reported here was more effective in repairing the sp^2^ network. Taking into account the relationship of *I*_D_/*I*_G_ with the extent of *π*-conjugation and the concentration of defects on GO, the nrGO-PEG obtained by this green reduction method showed more integrated *π*-conjugation network, which is favorable for loading much more aromatic molecules via *π*-*π* stacking.

### Photothermal effect and biocompatibility

**Figure 4:**
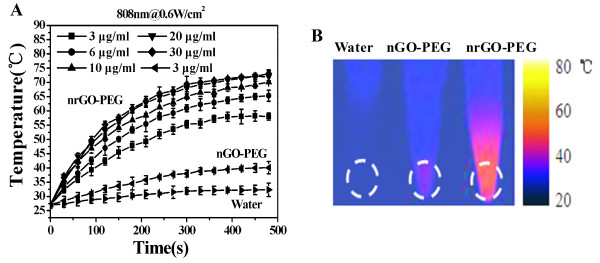
**Photothermal effect of nGO-PEG and nrGO-PEG. (A)** Temperature curves versus time during irradiation with 808 nm laser (0.6 W/cm^2^) for vials containing water, nGO-PEG (3 μg/mL), and various concentrations of nrGO-PEG, respectively. **(B)** Thermal images of vials with water, nGO-PEG, and nrGO-PEG (3 μg/mL) after irradiation by 808 nm laser (0.6 W/cm^2^) for 8 min.

As seen in Figure 4A, the temperature of vials containing 3, 6, 10, 20, and 30 μg/mL of nrGO-PEG solution readily reached to approximately 55°C, 60°C, 65°C, 68°C, and 70°C, respectively, within 5 min of 808 nm laser irradiation (0.6 W/cm^2^), displaying a concentration-dependent rapid photothermal heating at low nrGO-PEG concentrations (from 3 to 20 μg/mL). In strong contrast, under the same laser irradiation condition, 3 μg/mL of nGO-PEG solution remained below 40°C (Figure 4A). Figure 4B showed the corresponding thermal images of vials containing water, 3 μg/mL of nGO-PEG solution and 3 μg/mL of nrGO-PEG solution, respectively, after laser irradiation for 8 min. For the photothermal transferring efficiency [45], 28°C increase for 0.5 mL of solution with 3 μg/mL of nrGO-PEG was estimated to be approximately 59 J which amounts approximately to 32.7% of the irradiation laser energy (0.6 W/cm^2^, 5 min). However, the photothermal transferring efficiency of the 3 μg/mL of nGO-PEG solution was only about 11.7%. With the increase of nrGO-PEG concentration, 20 μg/mL of nrGO-PEG solution had about 50% transferring efficiency. Therefore, the nrGO-PEG obtained by this green reduction approach may be a great photosensitizer for PTT.

**Figure 5:**
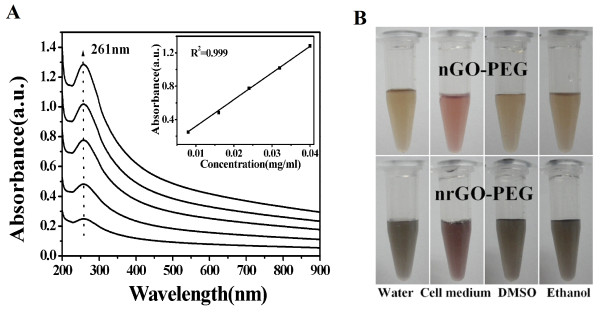
**Dispersibility of nrGO-PEG. (A)** UV-vis absorption spectra of the nrGO-PEG aqueous dispersion. Inset: correlation of absorbance at 261 nm against concentration. **(B)** Photographs of nGO-PEG and nrGO-PEG solution in several solvents after storage at 4°C for 3 months.

UV-vis spectrometer was also used to verify the formation of stable nrGO-PEG suspension (Figure 5A). If a homogeneous solution is formed, the absorbance at the characteristic peak should be in a linear relationship with the concentration on the basis of Beer's law [46]. We found that there was a good linear relationship (with *R*^2^ = 0.999) between the absorbance at 261 nm and the concentration of nrGO-PEG (Figure 5A inset), thus evidenced the great dispersibility of nrGO-PEG in water. It was also noted that the concentration of nrGO-PEG in water can be easily reached up to approximately 1.5 mg/mL. We also evaluated the dispersions of nGO-PEG and nrGO-PEG in cell medium contained 10% of fetal bovine serum and two kinds of common organic solvents (DMSO and ethanol) and found that the nrGO-PEG remained stable after storage in these solvents for more than 3 months at 4°C (Figure 5B), demonstrating that this facile and green reduction route did not disrupt the dispersion of nrGO-PEG, likely due to the presence of partial covalent PEG chains on nrGO-PEG. Because of the great biocompatibility under high concentration and the long-term stability in water, the nrGO-PEG obtained by this green reduction approach may become a promising biomaterial.

Recently, Robinson and coworkers reported a chemical reduction method for nGO-PEG by using hydrazine monohydrate as reduction reagent [20]. The nrGO-PEG obtained by this chemical reduction method afforded approximately 6.5-fold increase in NIR absorbance and 26°C increase under 5 min of 808 nm laser irradiation (0.6 W/cm^2^), but it aggregated in the solution due to the removal of functional groups from GO sheet [20]. Therefore, the nrGO-PEG obtained by this chemical reduction approach must be resuspended by functionalized PEG to increase its biocompatibility for bioapplication [20,21]. In contrast, the nrGO-PEG obtained by the present green reduction approach had not only approximately 7.6-fold increment in NIR absorbance and 43°C increase under the same laser irradiation (at 808 nm) (Figure 2B,C) but also very great biocompatibility (Figure 2B, C and 5).

Moreover, some natural extracts and nontoxic reagents including gelatin, NaOH, or KOH and alcohols have been also used to reduce GO [28,30,31]. Although these green reduction methods need low temperature (about 50°C to 100°C) and short react time (below 24 h), the obtained rGO showed relative bad stability in water (keep stable for less than 1 month). In contrast, the nrGO-PEG prepared with our green method could keep stable for at least 3 months (Figure 5), which is in favor of long-term bioapplication.

### Cellular uptake and *in vitro* photothermal therapy

**Figure 6:**
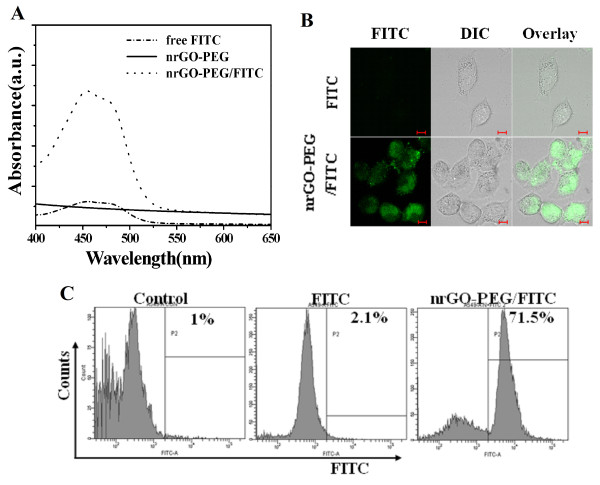
**Cellular uptake of nrGO-PEG in A549 cells. (A)** UV-vis absorbance spectra of free FITC, nrGO-PEG, and nrGO-PEG/FITC. **(B)** Fluorescence images of cells cultured with free FITC and nrGO-PEG/FITC, respectively. The adherent cells incubated with free FITC and nrGO-PEG/FITC, respectively, for 2 h were imaged by confocal microscope. **(C)** Cellular uptake ratio of nrGO-PEG. The cells were cultured with 100 μg/mL of nrGO-PEG/FITC and free FITC, respectively, for 2 h before FCM analysis.

To confirm the uptake of nrGO-PEG by tumor cells, FITC was used to label nrGO-PEG by physical adsorption. UV-vis spectra showed anapproximately 450 nm absorption peak (Figure 6A), demonstrating the binding of FITC to nrGO-PEG. A549 cells were then incubated with the complex nrGO-PEG/FITC for 2 h, and after removing the extracellular nrGO-PEG/FITC, the cells were observed by fluorescence microscopy. Much stronger fluorescence was seen inside the cells (Figure 6B), indicating the cellular uptake of nrGO-PEG. Furthermore, the cellular uptake ratio of nrGO-PEG was assessed by FCM analysis. As shown in Figure 6C, the cells cultured with 100 μg/mL of free FITC and nrGO-PEG/FITC had 2.1% and 71.5% of uptake ratio, respectively, indicating that liposoluble FITC nearly couldnot enter A549 cells, but nrGO-PEG particles could easily enter A549 cells likely via endocytosis. Highly accumulation of nrGO-PEG in tumor cells would enable selective photothermal heating and locally rapid temperature increase in tumor by 808 nm laser irradiation.

**Figure 7:**
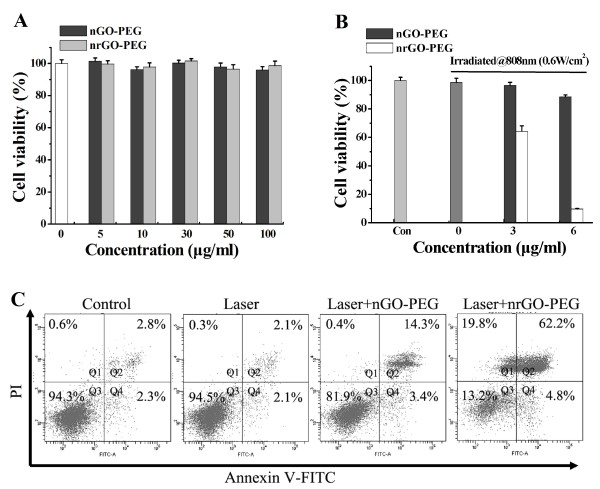
**Cytotoxicity of nGO-PEG and nrGO-PEG in A549 cells. (A)** Relative cell viability after treatment with different concentrations of nGO-PEG and nrGO-PEG for 24 h. **(B)** Relative cell viability after 808 nm laser irradiation (0.6 W/cm^2^) for 5 min for control cells and the cells cultured with different concentrations of nGO-PEG and nrGO-PEG. **(C)** FCM analysis of cell death induced by laser irradiation for control cells and the cells cultured with 6 μg/mL of nGO-PEG or nrGO-PEG for 2 h.

Toxicity is a common concern for all biomaterials. Exposure of A549 cells to 5, 10, 30, 50, and 100 μg/mL of nGO-PEG and nrGO-PEG, respectively, for 24 h did not induce a significant decrease of cell viability (Figure 7A), indicating that nGO-PEG and nrGO-PEG below 100 μg/mL were noncytotoxic to A549 cells.

To determine the cytotoxicity of nGO-PEG and nrGO-PEG under laser irradiation, A549 cells were incubated with various concentrations of nGO-PEG and nrGO-PEG solution for 2 hand then irradiated by 808 nm laser (0.6 W/cm^2^) for 5 min. CCK-8 assay showed that the laser irradiation did not induce significant cytotoxicity for control cells but induced approximately 35% decrease of cell viability for the cells incubated with 3 μg/mL of nrGO-PEG and approximately 90% decrease of cell viability for the cells incubated with 6 μg/mL nrGO-PEG (Figure 7B), further demonstrating the great PTT effect of nrGO-PEG. As control, the PTT effect of 3 and 6 μg/mL of nGO-PEG were remarkably lower than that of nrGO-PEG (Figure 7B). Moreover, FCM analysis with FITC Annexin V and propidium iodide staining was also used to examine the cell death induced by the photothermal effect of nGO-PEG and nrGO-PEG. As seen in Figure 7C, Q1 + Q2, Q3, and Q4 represented the regions of dead cells, living cells, and early apoptotic cells, respectively [8,47]. Consistent to the cytotoxicity assay (Figure 7B), laser irradiation did not induce significant cell death for control cells but induced remarkable cell death for the cells cultured with nGO-PEG or nrGO-PEG (6 μg/mL) for 2 h (Figure 7C). Moreover, after the laser irradiation, the cells cultured with nrGO-PEG presented much more dead cells compared with the cells cultured with nGO-PEG (Figure 7C). These *in vitro* results further demonstrate that the nrGO-PEG obtained by the present green reduction approach may be a great photosensitizer for PTT.

## Conclusions

In this work, we have developed a facile and green approach toward biocompatible and controlled reduction of nGO-PEG to nrGO-PEG solution. Covalent PEGylation not only prevents the aggregation of nGO but also dramatically promotes the reduction extent of nGO. This water-only reduction route is very convenient, nontoxic, and environment-friendly and has the ability to recover *π*-conjugation via repairing the postreduction defects. Moreover, the nrGO-PEG with approximately 50 nm in average size obtained by this green reduction approach has approximately 7.6-fold increment in NIR absorbance, great biocompatibility, and high cellular uptake ratio, making it a promising photoabsorbing agent for PTT comparable to carbon nanotubes and gold-based nanomaterials.

## Abbreviations

AFM: atomic force microscopy; FITC: fluorescein isothiocyanate; FTIR: Fouriertransform infrared spectroscopy; mPEG-NH2: methoxypolyethylene glycol amine; NIR: near-infrared; PEG: polyethylene glycol; PTT: photothermal therapy; rGO: reduced graphene oxide.

## Competing interests

The authors declare that they have no competing interests.

## Authors’ contributions

JC and TC conceived and designed the experimental strategy. JC performed the experiments and prepared the manuscript. TC and XW supervised the whole work and revised the manuscript. All authors read and approved the final manuscript.

## Authors’ information

Jingqin Chen and Xiaoping Wang are co-authors.
